# Switching from Natural Desiccated Thyroid to a Liquid Formulation of Levothyroxine for Hypothyroidism

**DOI:** 10.1155/2023/4252894

**Published:** 2023-12-28

**Authors:** Linda Khoshaba, Laurena Patarkatsi

**Affiliations:** Natural Endocrinology Specialists, Scottsdale, AZ, USA

## Abstract

Natural desiccated thyroid (NDT) is a treatment option for many patients with hypothyroidism, but some still exhibit symptoms despite achievement of normal levels of thyroid stimulating hormone (TSH). In this retrospective case series, 13 patients with hypothyroid symptoms were switched from NDT to a liquid formulation of levothyroxine (LT4; Tirosint®-SOL). Laboratory values ≥4 weeks following the switch showed a decrease in TSH levels, maintenance of free triiodothyronine (fT3) levels, and significant increases in free thyroxine (fT4) levels. Patients reported good tolerability, and case summaries are presented for four patients. In summary, this small retrospective case series showed that patients who still had hypothyroid symptoms despite use of NDT can respond well to oral LT4 liquid formulation, including patients who are intolerant of gluten and/or lactose or on hormone replacement therapy or iron supplementation.

## 1. Introduction

Hypothyroidism results from the deficiency of thyroid hormones, specifically when thyroid stimulating hormone (TSH) levels are elevated and free thyroxine (fT4) levels are reduced compared to reference ranges [1]. The most frequently reported clinical symptoms include fatigue, constipation, weight gain, dry skin, and intolerance to cold temperatures [1, 2]. Successful treatment of hypothyroidism not only focuses on the reduction of TSH levels but also the need for symptom resolution and improved quality of life (QOL). Administration of levothyroxine (LT4) is the recommended standard of care for the treatment of hypothyroidism according to joint guidelines from the American Thyroid Association and American Association of Clinical Endocrinologists [3, 4].

The use of natural preparations of thyroid hormone was the dominant form of all pharmacological treatment for hypothyroidism until the 1970s [5]. Many prescribers still utilize natural desiccated thyroid (NDT) [6, 7], derived from porcine thyroid glands, for the treatment of hypothyroidism. While these preparations contain both triiodothyronine (T3) and T4, they are not physiologically equivalent to those seen in humans [8] and possess potential variability in T3 and T4 concentrations [4]. Some positive effects of NDT treatment of hypothyroidism have been reported [9, 10], but NDT does not always improve QOL compared with LT4 treatment [11]. Due to their animal origin, NDT preparations are not an option for vegans, vegetarians, and those who avoid pork for religious reasons. Additionally, NDT preparations have not been widely tested in clinical trials, and as such, they lack evidence of their efficacy versus synthetic LT4. There are reports of adverse patient outcomes with compounded thyroid medications due to error [12] and deviations in potency among batches [13, 14]. Further, a United States Food and Drug Administration (FDA) inspection of the manufacturing facility for WP Thyroid® and Nature-Throid® in 2020 found that these products did not conform to standards for strength, purity, or quality [15] and thus were removed from the market. The FDA has not approved NDT preparations; however, they are regulated by the administration.

A liquid formulation of LT4 is available for the treatment of hypothyroidism. Unlike tablet formulations of levothyroxine and NDT preparations, this LT4 liquid formulation is composed only of levothyroxine, water, and glycerol [16] (Table 1) without other excipients that may interfere with absorption. As such, LT4 liquid formulation exhibits enhanced absorption [17–19] with a faster absorption rate [20]. In a retrospective study, patients who were switched from LT4 tablets to LT4 liquid formulation achieved a significant decrease in TSH levels within 60–90 days [21]. Patients treated with LT4 liquid formulation also remained within normal TSH ranges for a longer period of time compared with those on LT4 tablets [22] and exhibited significant improvements in QOL as measured on a validated, thyroid-specific questionnaire [23].

Although there are published reports of switching from LT4 tablets to NDT [9–11], to our knowledge, there is no published literature on switching from NDT to LT4 liquid formulation. Thus, this retrospective case series of patients with hypothyroidism evaluated those who had been treated with NDT but still experienced hypothyroid symptoms who were subsequently switched to LT4 liquid formulation.

## 2. Methods

This was a retrospective analysis of patients with a prior diagnosis or hypothyroidism or Hashimoto's disease who initiated treatment from December 2020 to May 2022 at Natural Endocrinology Specialists PLLC, Scottsdale, AZ, USA. At the initial visit, patients with hypothyroidism exhibited its associated symptoms despite prior treatment with NDT. Patients were switched to individually packaged monodose ampules of LT4 liquid formulation (Tirosint®-SOL, levothyroxine sodium oral solution, IBSA Pharma Inc., Parsippany, NJ), with the initial dose determined on an individual basis by weight [16] and clinical judgment. LT4 liquid formulation was to be taken upon rising in the morning (between 5:00 and 11:00 am) without food. Two patients were also treated with liothyronine. Data are presented for patients with both pre-switch and post-switch laboratory values for TSH, free T3 (fT3), and free T4 (fT4). Testing was performed by LabCorp or Quest Diagnostics, which utilized the electrochemiluminescence immunoassay for TSH and fT4 and equilibrium dialysis and high-pressure liquid chromatography/tandem mass spectrometry for fT3. Patients were encouraged to use the same testing laboratories for all measurements. LabCorp reference values were used: TSH, 0.45–4.5 *μ*IU/mL [24]; fT3, 2.0–4.4 pg/mL [25]; and fT4, 0.82–1.77 ng/dL [26]. Retrospective data were verified by both authors. Data are expressed as medians with interquartile ranges. The Wilcoxon matched-pairs signed rank test was used in GraphPad Prism (GraphPad Software, San Diego, CA, USA), version 9.4.1, to test for significant differences between pre-switch and post-switch to LT4 liquid formulation. *p* ≤ 0.05 was considered to be significant. Tolerability and adverse events were also noted at every visit.

## 3. Results

### 3.1. Patient Demographics

Thirteen patients were included in this analysis, all female (Table 2; Supplemental Table 1). Eight had a diagnosis of autoimmune thyroiditis (Hashimoto's disease) and five of hypothyroidism. Five patients had gluten intolerance or celiac disease, with three of those patients also reporting lactose intolerance. Four patients were on hormone replacement therapy. Patients were taking an average of 9 supplements (range 0–18), some of which were multivitamins and iron. Most (12/13) wanted to switch to LT4 liquid formulation because of continued thyroid symptoms despite use of NDT.

### 3.2. Laboratory Measures of Thyroid Function

**Figure 1:**
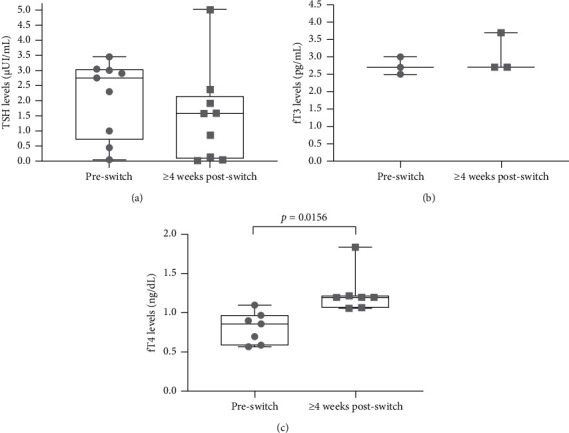
Box plots of (a) TSH values before and ≥4 weeks after the switch to LT4 liquid formulation for patients with TSH values within or below the normal range (0.45–4.5 *μ*IU/mL) before the switch. (b) fT3 values before and ≥4 weeks after the switch to LT4 liquid formulation for patients who did not also receive liothyronine. The normal range for fT3 values was considered to be 2.0–4.4 pg/mL. (c) fT4 values before and ≥4 weeks after the switch to LT4 liquid formulation. The normal range for fT4 values was considered to be 0.82–1.77 ng/dL. Median (line within box), first and third quartiles (top and bottom of box), and minimum and maximum values (whiskers) are indicated. Gray symbols show the individual data points. fT3, free triiodothyronine; fT4, free thyroxine; TSH, thyroid stimulating hormone.

Most patients were within the normal TSH range prior to the switch to LT4 liquid formulation (Figure 1(a) and Table 3). Despite these data, they presented with symptoms of hypothyroidism, including fatigue, hair loss, and weight gain (Table 2). Including only the patients receiving LT4 liquid formulation (and not also liothyronine) who had TSH values within or below the normal range, the overall median TSH values decreased from 2.75 *μ*IU/mL prior to the switch to 1.57 *μ*IU/mL ≥ 4 weeks after the switch to individualized doses of LT4 liquid formulation (Figure 1(a)). For all patients, the pre-switch median TSH values were 2.90 *μ*IU/mL, with a post-switch median of 1.89 *μ*IU/mL. For all patients except for the 2 who were also receiving liothyronine, the median TSH values of pre-switch and post-switch were 2.9 and 1.58 *μ*IU/mL, respectively.

Patient K had TSH levels within the normal range before the switch to LT4 liquid formulation 25 *μ*g but levels increased following the switch. This patient had also initiated estrogen treatment, which also affected TSH values. The dosage of LT4 liquid formulation was increased, but no further follow-up data were available for this patient.

Three patients (A, G, M) had lower than normal TSH values following the switch, and all had their LT4 liquid formulation dosages decreased. Follow-up data were available for one patient (A) after the dose decrease, which showed that this patient still had TSH levels below the normal range at the next assessment, so the dose was further decreased.

Three patients (B, F, J) exhibited levels of TSH higher than the upper limit of the normal range of 4.5 *μ*U/mL prior to the switch to LT4 liquid formulation (Table 3). Of these, TSH levels in two of these patients (B, J) decreased to fall within the normal range within ≥4 weeks following the switch. The third of these patients (F) had abnormally high TSH levels prior to the switch that decreased from 9.82 to 7.40 *μ*IU/mL at day 35 following the switch to LT4 liquid formulation 100 *μ*g and the addition of liothyronine 10 *μ*g. With a dose increase in the LT4 liquid formulation to 112 *μ*g (skipping the dose one day per week) and continuation of liothyronine 10 *μ*g, the patient achieved a TSH level of 2.11 *μ*IU/mL at 40 weeks following the initial switch to LT4 liquid formulation and reported an improvement in her health.

Data for fT3 values for before and after the switch were available for three patients (Figure 1(b)). Prior to the switch, all patients had fT3 values within the normal range (median 2.7 pg/mL), and all maintained normal values following the switch to LT4 liquid formulation (median 2.7 pg/mL).

Seven patients had data for fT4 values before and after the switch to LT4 liquid formulation (Figure 1(c)). The fT4 values for three patients (E, F, K) were below the normal range, and the other four patients' values were within the normal range prior to the switch. All patients' fT4 values increased with the switch to LT4 liquid formulation, from a median of 0.86 to 1.20 ng/dL, *p*=0.0156.

### 3.3. Tolerability and Adverse Events

For all follow-up visits in which tolerability was recorded, tolerability was described as “good,” “great,” or “did not notice any difference from NDT.” Patient B reported loose stools at the fourth visit (but notably had a history of acute gastroenteritis), when her LT4 liquid formulation dose was decreased (see patient summary below). Patient G, who had a decrease in TSH levels to 0.01 following the switch to LT4 liquid formulation, reported good tolerability but also loose stools on day 67 post-switch. The dose of LT4 liquid formulation was decreased from 150 to 112 *μ*g, but no further follow-up data were available.

### 3.4. Patient Summaries

Case summaries are presented for select patients.

Patient B was a female, aged 47 years with a BMI of 32.5 kg/m^2^, hypothyroidism, and latent autoimmune diabetes of adults, though she was noncompliant with diabetes medications since they were not covered by her insurance. The patient reported fatigue and allergies to dairy and gluten. The patient's initial visit to the clinic was for the reestablishment of care. The patient was not taking any thyroid medication at the time of the initial visit but had previously taken WP Thyroid and then switched to NP Thyroid 2.5 grain (150 mg). TSH levels were elevated (6.63 *μ*IU/mL) prior to the switch to LT4 liquid formulation 150 *μ*g (once in the morning, 30 minutes away from food) and decreased to 1.91 and 0.801 *μ*IU/mL at 30 and 60 days post-switch, respectively. The patient maintained fT3 levels within the normal range prior to and 30 days after the switch (both 2.7 pg/mL), and fT4 levels increased from pre-switch to day 30 post-switch, from 0.86 to 1.2 ng/dL. The patient did not notice any difference in tolerability between NDT and LT4 liquid formulation. At day 59 post-switch, the dose of LT4 liquid formulation was increased to 175 *μ*g but was adjusted back to 150 *μ*g when the patient reported loose stools, gastroenteritis from the prior day, and good tolerability to LT4 liquid formulation at day 80 post-switch. At day 150 post-switch, the patient had TSH levels of 3.44 *μ*IU/mL, no longer had loose stools, and reported a 5-pound weight loss and that she was overall satisfied with her treatment.

Patient D was a female, aged 41 years with a BMI of 17.9 kg/m^2^, on 12 supplements, and with no known allergies at the initial visit. Her reasons for the initial visit were Hashimoto's thyroiditis, general fatigue, and adrenal issues. She reported an inability to gain weight. The patient had taken compounded thyroid medication but felt horrible, so she switched to NP Thyroid and then to Armour Thyroid. TSH levels prior to the switch to LT4 liquid formulation 125 *μ*g were 3.05 *μ*IU/mL and decreased to 0.853 *μ*IU/mL at day 83 following the switch. She also received intravenous iron due to low (15 ng/mL) ferritin levels. At the sole follow-up visit after the switch, the patient reported considerable improvement in symptoms and an 8-pound (3.6 kg) weight gain since the first visit. She expressed satisfaction with her treatment and wished she had started LT4 liquid formulation sooner.

Patient C was a female, aged 64 years with a BMI of 28.9 kg/m^2^, and had a history of autoimmune thyroiditis. She was taking five supplements as well as hormone replacement therapy for menopause (estradiol, progesterone, and testosterone) and presented with abnormal weight gain, hyperlipidemia, essential fatty acid deficiency, and erythrocyte abnormality. The reason for the initial visit was to reestablish care and receive a yearly physical. This patient was taking NP Thyroid 90 mg and was switched to LT4 liquid formulation 150 *μ*g. At day 29 following the switch, TSH levels were 1.91 *μ*IU/mL (from a pre-switch value of 3.0 *μ*IU/mL). Reported tolerability to LT4 liquid formulation was “good” at the visit 57 days post-switch, and the dose was increased to 175 *μ*g. At day 89 post-switch, TSH values were 2.14 *μ*IU/mL, and the patient was tolerating the dose well as reported at the day 98 visit. The patient maintained fT3 levels within the normal range, with values of 3.0, 2.7, and 2.7 pg/mL prior to the switch and at days 29 and 89 post-switch, respectively. There was an increase in fT4 levels with treatment with LT4 liquid formulation, with 1.1 ng/dL pre-switch, 1.2 ng/dL at day 29 post-switch, and 1.4 ng/dL at day 89 post-switch.

Patient J was a female, aged 65 years, with a BMI of 19.9 kg/m^2^, and had a history of autoimmune thyroiditis. Allergies to gluten and dairy, among others, were reported. The patient was on 14 supplements and presented with menopause, fatigue, nutritional anemia, hyperlipidemia, osteopenia, migraines, allergies, hair loss, and difficulty sleeping. She had taken Armour Thyroid but was unable to tolerate it and so had switched to NP Thyroid. TSH levels were above normal at 8.66 *μ*IU/mL prior to the switch to LT4 liquid formulation 125 *μ*g and decreased to 2.41 *μ*IU/mL 38 days following the switch. The patient sent a message 5 days following the switch that she was pleased with LT4 liquid formulation. At the visit on day 56 post-switch, the patient reported continued hormonal issues and was receiving testosterone and estradiol injections. Tolerability of LT4 liquid formulation was reported as “good,” and the dose was changed at this visit to 125 *μ*g plus 13 *μ*g for a total of 138 *μ*g daily. Since TSH levels decreased to 0.410 *μ*IU/mL at day 104 post-switch, dosing of LT4 liquid formulation was changed to at the next follow-up visit (119 days post-switch) to 125 *μ*g plus 13 *μ*g only on Saturdays and Sundays, with 125 *μ*g on Mondays through Fridays. Tolerability of LT4 liquid formulation was again reported as “good” at this visit.

## 4. Discussion

In this retrospective review, most patients had TSH levels within the normal range prior to the switch to LT4 liquid formulation yet remained symptomatic. Here, these patients generally achieved a further decrease in TSH levels following the switch to LT4 liquid formulation. In prior studies, patients with initially normal TSH values who were switched to LT4 liquid formulation from LT4 tablets have also shown significant decrease in TSH levels [21, 27].

Although 0.45–4.5 *μ*IU/mL is the normal range of values for TSH, the optimal upper limit has been proposed to be 2.5 *μ*IU/mL since the TSH levels of >95% of euthyroid individuals are below that value [28]. In their clinical experience, the authors have found an optimal TSH range of 1-2. The median TSH values decreased from 2.75 *μ*IU/mL prior to the switch to 1.57 *μ*IU/mL following the switch to LT4 liquid formulation in patients whose TSH levels were within or below the normal range prior to the switch.

Prior literature has described cases in which comorbid conditions, including lactose intolerance, celiac disease, chronic atrophic gastritis, and bowel resection surgery [29], as well as pregnancy [30], affect the ability to absorb LT4. Several patients (A, B, E, F, J) in this study had intolerances to gluten and/or lactose and had sensitivities to other foods (C, I, L), which are known to decrease the absorption of LT4. Seven of these patients were able to decrease their TSH levels by switching to the LT4 liquid formulation (the eighth patient had TSH levels within the normal range before and after the switch). Notably, three of the patients with gluten and/or lactose intolerance (B, F, J) had elevated pre-switch TSH levels, supporting the idea that they were unable to properly absorb T4. Liquid LT4 has previously ameliorated impaired absorption issues, including in 8 patients with lactose intolerance whose TSH levels normalized when switched from LT4 tablets to LT4 liquid formulation [17]. Further, a case study reported that symptoms resolved and TSH levels stabilized in a patient with Hashimoto's thyroiditis and celiac disease following the switch from LT4 tablets to LT4 liquid formulation and a gluten-free diet [31]. Another case study found that switching a patient with Hashimoto's disease, gastroparesis, and small intestinal bacterial overgrowth to LT4 liquid formulation from compounded T4/T3 achieved TSH levels within the optimal range with resolution of symptoms [32].

The concomitant use of certain drugs and supplements, including those containing calcium, iron, biotin, or any multivitamin, can also interfere with the absorption of LT4 [29]. In addition to noncompliance with thyroid medication and timing of supplements, a variety of factors can affect the ability to achieve TSH values within the normal range, including the administration of LT4 with food as well as consuming large quantities of fiber, papaya, or coffee [29]. Caffeine can also affect serum levels of TSH, fT3, and fT4 for several hours following consumption [33]. Several patients (A, D, F, I, L) were taking iron supplements or received intravenous iron. Four of these patients exhibited decreases in TSH levels following the switch to LT4 liquid formulation, with the fifth maintaining TSH levels in the normal range, indicating that absorption of LT4 liquid formulation was not affected by the supplemental iron. Previous prospective studies showed decreases in TSH levels following the switch from LT4 tablet to LT4 liquid formulation in patients with hypothyroidism who were also taking iron supplements [34], multiple interfering drugs [18], or proton pump inhibitors [19]. The four patients on hormone replacement therapy in the present study were also able to maintain TSH levels in the normal range.

There is no scientifically validated algorithm to switch patients from LT4 monotherapy to NDT, leading to potential overdosing in some patients. It is noteworthy that in this retrospective analysis, levels of fT3 were maintained within the normal range following the switch from NDT to the monotherapy LT4 liquid formulation. These cases demonstrated the ability of the LT4 liquid formulation to maintain normal levels of serum T3, which is of great biological importance [35]. In addition, this finding suggests that conversion of LT4 to LT3 was not an issue for the patients in this study. Only two patients (E, F) in this study received concomitant liothyronine (T3); both had celiac disease and a history of unsuccessful use of NDT and compounded thyroid medication. While NDT preparations contain both T3 and T4, the present study demonstrates that T3 supplementation was not necessary to maintain fT3 levels in most patients receiving LT4 liquid formulation monotherapy. Additionally, none of the patients in this study asked to be prescribed T3 once they had switched to LT4 liquid formulation. As expected, treatment with LT4 liquid formulation resulted in significant increases in fT4 levels consistent with previous observations with LT4 liquid formulation compared with LT4 tablets in patients with hypothyroidism [23]. Further, the fact that three patients (A, G, M) had lower than normal TSH values following the switch shows that a conservative approach to treatment is best due to the rapid absorption of the LT4 liquid formulation. From a clinical perspective, when switching to LT4 liquid formulation, the availability of a wide range of dosing options helps to prevent overprescribing, development of hyperthyroidism, and adverse effects patients may experience with overtreatment. Furthermore, treating with monotherapy allows the patient to be on the minimal amount medication with the greatest benefit.

Overall analysis of the use of LT4 liquid formulation showed that it was well tolerated by all patients in the study. Patients B and G reported loose stools, a known adverse reaction of overdosage of LT4 [16], following the switch (though patient B reported concomitant gastroenteritis and had diabetes, which is known to cause gastrointestinal issues, including diarrhea [36]). Their doses of LT4 liquid formulation were subsequently decreased, and patient B reported no loose stools at the next follow-up visit. Additional follow-up data were unavailable for patient G, but both patients reported good tolerability of LT4 liquid formulation.

The limitations of this study are due to its retrospective nature. Future studies could be prospective with more uniform timing of laboratory assessments and should include a QOL questionnaire to better capture the effects on daily life of LT4 liquid formulation treatment of hypothyroidism. In addition, some patients in this study had their assessments performed at a different laboratory and/or at different times of day, which may have led to some differences in laboratory values.

This first-of-its-kind study examined switching from NDT to LT4 liquid formulation, a monotherapy free from excipients, in patients with hypothyroidism. Treatment with LT4 liquid formulation led to maintenance or normalization of laboratory values, resolution of hypothyroid symptoms, and good tolerability. When dose adjustments were required, they were mainly downward titrations, which were most likely needed due to the enhanced absorption of LT4 liquid formulation. No patient switched back to NDT because of worsening symptom control, and all patients were satisfied with treatment. These cases demonstrate that LT4 liquid formulation can be part of a treatment plan to holistically address the common issues experienced by patients with hypothyroidism.

## Figures and Tables

**Table 1:** Excipients in commercially available thyroid treatments.

Excipient	Branded or generic LT4 tablets	Natural dessicated thyroid	Liquid LT4 formulation
Animal-sourced ingredients		X	
Corn starch	X		
Dyes	X		
Gluten	X^*∗*^		
Glycerol			X
Lactose	X	X^*∗*^	
Magnesium or calcium stearate	X	X	
Microcrystalline cellulose		X^*∗*^	
Povidone	X		
Sodium starch glycolate	X	X^*∗*^	
Sucrose or dextrose	X	X	
Talc	X		
Water			X

^*∗*^In some formulations.

**Table 2:** Baseline patient demographics and disease characteristics.

Patient	Sex	Age (years)	BMI (kg/m^2^)	Primary thyroid diagnosis	Comorbidities	Prior thyroid treatment	Medications/supplements	Reason for switch	Allergies	Symptoms
A	F	59	17.9	(i) Autoimmune thyroiditis	12, including celiac disease	WP Thyroid 2 grain (130 mg) and then switched to NP Thyroid 2 grain (120 mg) due to WP Thyroid recall	13, including ferrochel (iron chelate)	Symptoms	(i) Sulfur (ii) Diladon (iii) Gluten	(i) Abdominal pain (ii) Nausea (iii) Menopausal symptoms of hot flashes and night sweats

B	F	47	32.5	(i) Hypothyroidism (ii) Multinodular goiter	11	WP Thyroid and then switched to NP Thyroid 2.5 grain (150 mg)	5	Symptoms	(i) Sulfa drugs (ii) Penicillin (iii) MSG (iv) Wheat (v) Dairy	(i) History of thyroid disorder (ii) Noncompliant with diabetes medications (iii) Hot flashes (iv) Abdominal flatulence

C	F	64	28.9	(i) Autoimmune thyroiditis (ii) Hypothyroidism (iii) Nontoxic goiter	6	NP Thyroid 90 mg (1.5 grains)	6	Symptoms	None known	(i) History of hypothyroidism

D	F	41	17.9	(i) Autoimmune thyroiditis (Hashimoto's disease)	6	Armour Thyroid 90 mg	12, including a multivitamin with iron	Symptoms	None known	(i) Fatigue (ii) Moodiness (iii) Irritability (iv) Depression

E	F	49	19.9	(i) Hypothyroidism	7, including celiac disease	Armour Thyroid, then NP Thyroid, and then compounded thyroid medication	15	Symptoms	(i) Ciprofloxacin (ii) Weeds (iii) Trees (iv) Grasses	(i) Fatigue (ii) Irregular periods (iii) Allergies

F	F	60	23	(i) Autoimmune thyroiditis (Hashimoto's disease)	5, including celiac disease	NDT and then compounded levothyroxine/liothyronine 90/25 *μ*g	18	Symptoms	(i) Sulfa (ii) Gluten (iii) Dust (iv) Lactose	(i) History of Hashimoto's disease

G	F	56	26.5	(i) Hypothyroidism	5	Nature Thyroid 1.5 grain	8	Symptoms	(i) Naproxen (ii) Eggs (iii) Almonds (iv) Bananas	(i) Hypothyroidism (ii) Constipation (iii) Fatigue (iv) Weight gain

H	F	65	N/A	(i) Hypothyroidism	5	NP Thyroid 60 mg	10, including multivitamin with iron	Symptoms	(i) Sulfa drugs	(i) Stress (ii) Hives (iii) Hair loss

I	F	53	N/A	(i) Autoimmune thyroiditis (Hashimoto's disease)	6	WP Thyroid and then Synthroid and T3 sustained release	13, including iron	Symptoms	(i) Cefalexin (ii) Epinephrine (iii) Adhesives (iv) Iodine contrast	(i) Menopause (ii) Autoimmunity (iii) GI concerns

J	F	65	19.9	(i) Autoimmune thyroiditis	7	Armour Thyroid and then NP Thyroid 90 mg = 1.5 grains	12	Symptoms	(i) Penicillin (ii) Dairy (iii) Gluten (iv) Soy (v) Mold	(i) History of Hashimoto's disease (ii) Hair loss (iii) Difficulty sleeping

K	F	30	24	(i) Autoimmune thyroiditis	5	NP Thyroid 90 mg = 1.5 grains	None	Symptoms	None known	(i) Skin issues (ii) History of hypothyroidism (iii) Menopause symptoms (post-hysterectomy) (iv) Severe joint pain, swelling (v) Hair loss

L	F	43	N/A	(i) Hypothyroidism	5	NDT, Synthroid	3, including iron chelate	Intolerance of NDT	(i) Coconut (ii) Sulfa drugs	(i) Weight gain (ii) Fatigue (iii) Hormonal imbalance

M	F	55	N/A	(i) Autoimmune thyroiditis	11	NP Thyroid 120 mg	7	Symptoms	None known	(i) Metabolic syndrome (ii) Menopause

N/A, not available. A full list of comorbidities and medications/supplements is available in Supplemental Table 1.

**Table 3:** TSH values before the switch and ≥4 weeks after the switch to LT4 liquid formulation for all patients.

Patient	TSH (*μ*U/mL)	
	Before switch	≥4 weeks after switch
A	2.75	0.032
B	6.63	1.91
C	3.0	1.91
D	3.05	0.0853
E	0.605	1.89
F	9.82	7.40
G	0.44	0.01
H	0.993	1.58
I	3.45	1.57
J	8.66	2.41
K	2.3	5.01
L	2.9	2.37
M	0.04	0.13

The normal range for TSH values was considered to be 0.45–4.5 *μ*IU/mL.

## Data Availability

Data are available upon request to the corresponding author.

## Supplementary Materials

Supplementary Materials Supplemental Table 1: patient comorbidities and medications/supplements at baseline.
