# Supplementary material for: Switching from Natural Desiccated Thyroid to a Liquid Formulation of Levothyroxine for Hypothyroidism
Source: Case Rep Endocrinol. 2023 Dec 28;2023:4252894. doi: 10.1155/2023/4252894 (PMC10766474; doi:10.1155/2023/4252894)
Supplement: Supplementary Materials — Supplemental Table 1: patient comorbidities and medications/supplements at baseline. [file 4252894.f1.docx]

**Supplemental Table 1.** Patient comorbidities and medications/supplements at baseline.

| **Patient** | **Age, years** | **BMI, kg/m^2^** | **Primary thyroid diagnosis** | **Comorbidities** | **Medications/**  **supplements** |
| --- | --- | --- | --- | --- | --- |
| A | 59 | 17.9 | • Autoimmune thyroiditis | • Malabsorption  • Fatigue  • Hyperlipidemia, unspecified  • Celiac disease  • Menopausal and female climacteric states  • Hypercalcemia  • Dorsalgia, unspecified  • Other abnormal glucose  • Eosinophilia, unspecified  • Elevated Lipoprotein (a)  • Cervicalgia  • Abnormal results of kidney function studies | • Hormone replacement therapy  • Red Yeast Rice  • Coenzyme Q10  • Ferrochel (iron chelate)  • Omega-3 fatty acids  • B Supreme (B Max)  • Vitamin D  • Ca/Mg  • A-Drenal  • Liquid Zinc  • Elderberry  • Liquid B Max  • Probiotic |
| B | 47 | 32.5 | • Hypothyroidism  • Multi-nodular goiter | • Type 1 DM  • Stress  • Elevated hsCRP  • Overweight  • Fatigue  • Other specified menopausal and perimenopausal disorders  • Hyperlipidemia  • Abdominal pain  • LADA  • Autoimmunity  • Stress | • Insulin degludec  • Insulin lispro  • Annatto Synergy  • Vitamin D  • Silver spray |
| C | 64 | 28.9 | • Autoimmune thyroiditis  • Hypothyroidism  • Non-toxic goiter | • Abnormal weight gain  • Hyperlipidemia  • Menopause  • Essential fatty acid deficiency  • Other abnormality of red blood cells  • Stress | • Hormone replacement therapy: estradiol, progesterone, testosterone  • Zinc  • Vitamin D3  • Pure Nature  • DHEA  • Quercetin |
| D | 41 | 17.9 | • Autoimmune thyroiditis (Hashimoto’s disease) | • Chronic Infections  • Estrogen Dominance  • Hyperlipidemia  • Vitamin D deficiency  • Insomnia  • Weight loss | • Baby and Me 2 prenatal vitamin  • Ashwagandha  • Vitamin D3 + K1 and K2  • Pro EPA 850 EPA/200 DHA  • Selenium  • Vimergy Zinc  • Rodiola  • 5-HTP  • Vitamin B12 and Methyl Folate  • Magnesium glycinate  • Be Serene Natural Anxiety Relief  • Progesterone |
| E | 49 | 19.9 | • Hypothyroidism | • Polymenorrhea  • Allergies  • Hyperprolactinemia  • Acne  • Osteopenia  • Celiac disease  • Sleep apnea | • X factor Plus  • L-glutathione  • Vitamin C  • Bone and Joint  • Vitamin D  • Zinc  • BioCleanse  • Vital Biome  • ProBio 5  • Mega  • Cortisol  • Calm Ease  • Monolaurin  • Prolan H  • Milk thistle |
| F | 60 | 23 | • Autoimmune thyroiditis (Hashimoto’s disease) | • Anxiety  • Depression  • Celiac disease  • History of endometriosis  • Vitamin D deficiency | • 4Sight  • D Hist  • Biotics MCS-2  • Orthobiotic  • Fen-GRe  • K Force  • Psyillum husk  • Tripel EFA guard  • Melatonin  • Immunplex  • Perquie Endura  • b Vital (Maca and Deer Antler)  • Symplex F  • Selenium  • Liquid droppers- Neem  • INF Fighter  • Echinacea  • Gingko |
| G | 56 | 26.5 | • Hypothyroidism | • Chronic constipation  • Chronic rhinitis  • Anemia  • Vitamin D deficiency  • Weight gain | • Vitamin C  • SuperFolate  • Vitamin D  • Magnesium citrate  • Magnesium glycinate  • Bottom's Up Balm  • D-Hist  • Influenzinum |
| H | 65 | N/A | • Hypothyroidism | • Chronic inflammatory demyelinating polyradiculoneuropathy  • Urticaria  • Megaloblastic anemia  • Iron deficiency anemia  • Paresthesia of skin | • Hormone Protect (Xymogen)  • Cortisolv (Xymogen)  • Adrenal Manager (Xymogen)  • B Active (Xymogen)  • Zinc  • Turmeric (Qunol)  • Multivitamin with iron  • Omega Oil Mono 650  • Probiotic  • K2 D3 5000 |
| I | 53 | N/A | • Autoimmune thyroiditis (Hashimoto’s disease) | • Menopause  • Insomnia  • Stress  • Iron deficiency anemia  • Vitamin D deficiency  • Raised antinuclear antibody titer | • EstroGel  • Prometrium  • Relora  • Pro Omega  • Black cumin seed oil  • Vitamin B12  • Vitamin D  • Magnesium glycinate  • L-Lysine  • Vitamin C  • Colostrum  • Iron  • DIM |
| J | 65 | 19.9 | • Autoimmune thyroiditis | • Menopause  • Fatigue  • Nutritional anemia  • Hyperlipidemia  • Osteopenia  • Migraines  • Allergies | • Selenium  • Children's ENT-Pro 2 Billion  • Berberine  • Vit-A  • Zinc picolinate  • Panplex 2-phase  • LipoZome-C 1000 mg/Vit-B12  • Trizomal Glutathione  Immunopad  • San*Qi  • Ganolegro  • Bronca.Wise  • Baicalin |
| K | 30 | 24 | • Autoimmune thyroiditis | • Menopause  • Stress  • Psoriasis  • Joint pain  • Urinary tract infection | None |
| L | 43 | N/A | • Hypothyroidism | • History of infertility  • Food allergies  • Fatigue  • Abnormal glucose  • Irregular menstruation | • Iron chelate  • Vitamin D  • UT synergy |
| M | 55 | N/A | • Autoimmune thyroiditis | • Myalgia  • Hyperlipidemia  • Menopause  • Obesity  • Abnormal liver enzymes  • Androgen excess  • Vitamin D deficiency  • Rosacea  • Elevated C Reactive protein  • Gout  • Abnormal glucose | • Pro Omega  • Vitamin D  • Quercetin  • Zinc  • B-Complex  • N-acetyl cysteine  • CoQ 10 |

5-HTP, 5-Hydroxytryptophan; DHEA, dehydroepiandrosterone; LADA, latent autoimmune diabetes of adults; N/A, not available.
